# Supplementary figures and images for: Proinsulin degradation and presentation of a proinsulin B-chain autoantigen involves ER-associated protein degradation (ERAD)-enzyme UBE2G2
Source: PLoS One. 2024 May 24;19(5):e0287877. doi: 10.1371/journal.pone.0287877 (PMC11125532; doi:10.1371/journal.pone.0287877)

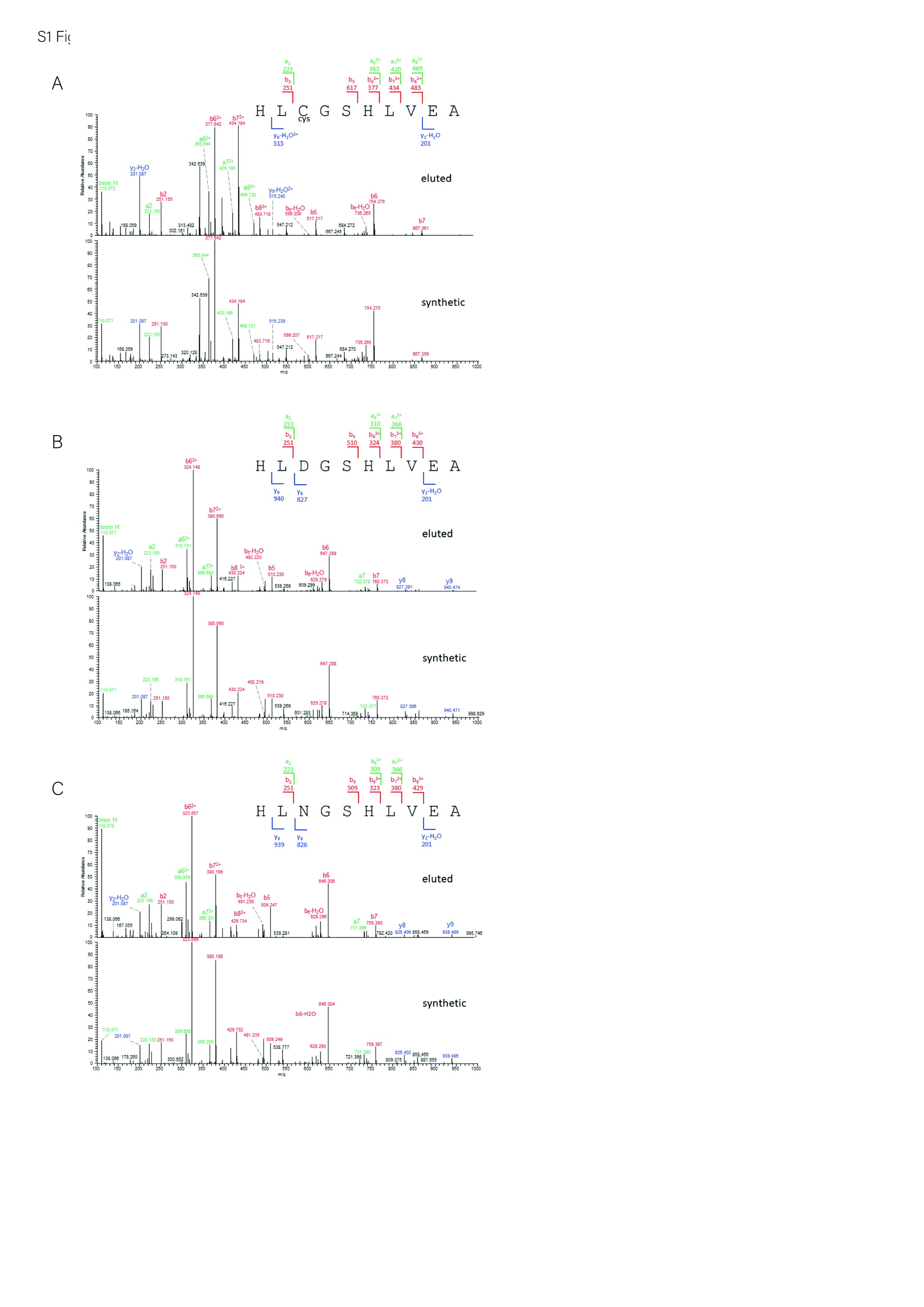

Supplement: S1 Fig — (A-C) (Top) Shown are mass spectrometry spectra of WT (A) or mutant (B, C) proinsulin-derived peptides, indicating the identification of proinsulin peptides as eluted from (mutant) proinsulin expressing cells shown in Figs 5 and 6. (Bottom) spectra show measurements of corresponding peptides for validation. (TIF) [file pone.0287877.s001.tif]

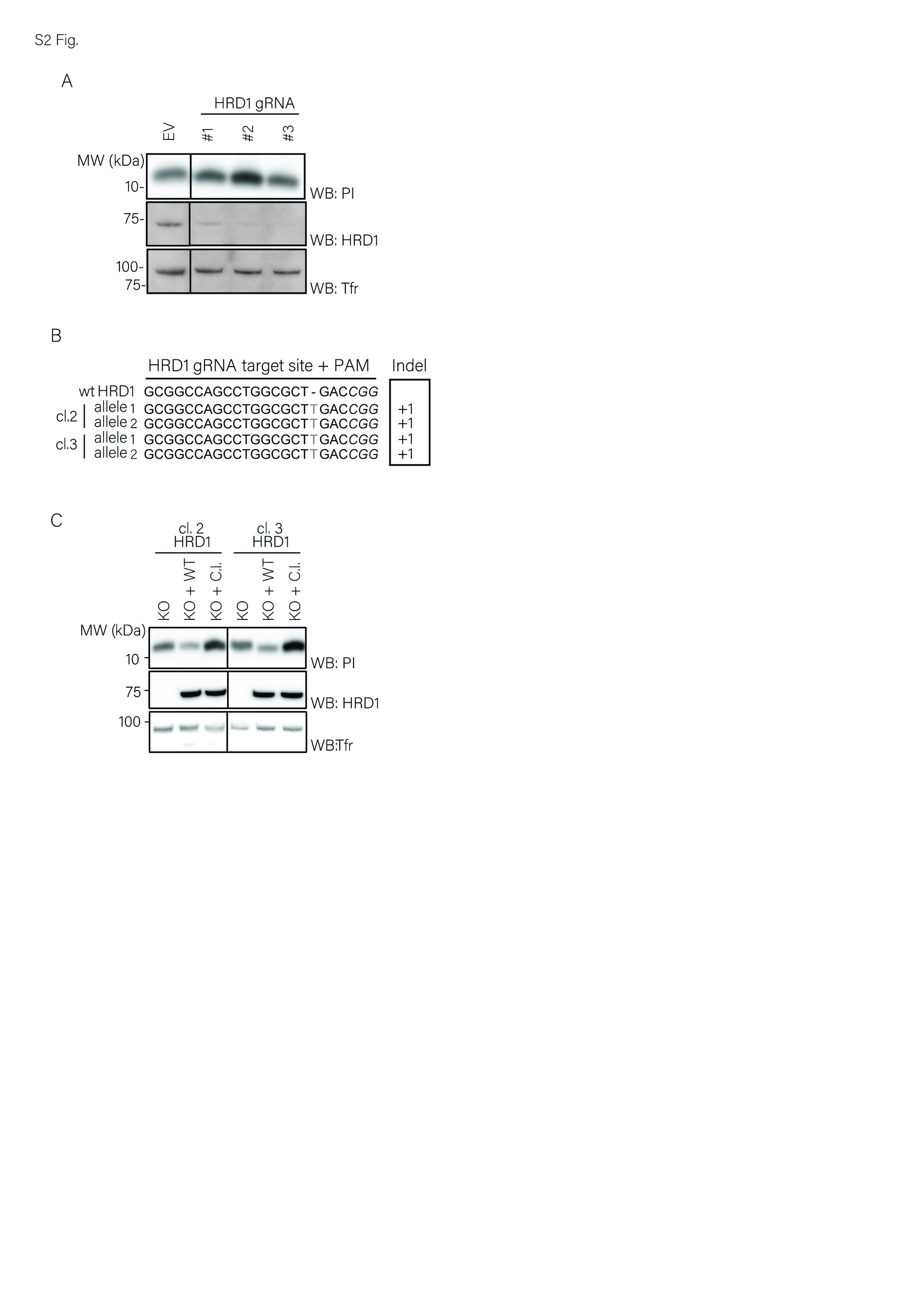

Supplement: S2 Fig — A. K562 cells stably expressing HLA-A*02:01 and PPI were transduced with three different CRISPR-Cas9 vectors containing a gRNA sequence directed against the N-terminal region of the HRD1 gene and selected with puromycin. Polyclonal cell populations were analyzed for HRD1 and proinsulin levels by Western blotting. B. Monoclonal knockout cell lines were generated by limiting dilution of gRNA#3 polyclonal cells shown in (A). Genomic DNA was isolated and sequenced for the presence of deletions within the gRNA target region. Both HRD1 alleles of clones 2 and 3 were aligned to a reference sequence (NCBI gene entry 84447). C. K562 cells from (B) were retransduced with an empty cDNA vector (KO) or a cDNA vector encoding HRD1 (WT), or a catalytically inactive mutant (C1A). Cells were sorted on mAmetrin expression to obtain a pure population. Next cell lysates were analyzed by WB for expression of HRD1 and proinsulin. Human transferrin receptor was used as a loading control. (TIF) [file pone.0287877.s002.tif]

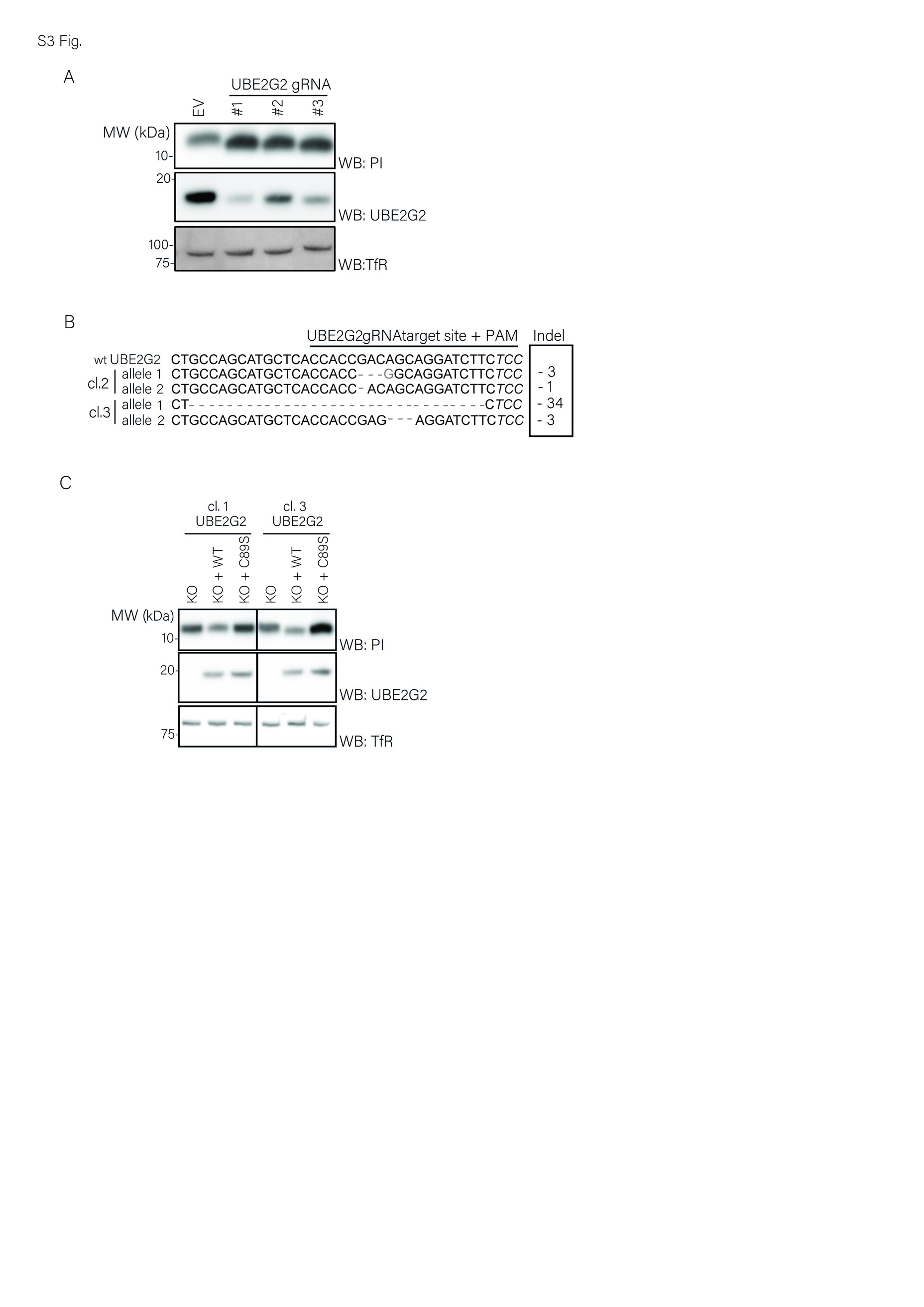

Supplement: S3 Fig — A. K562 cells stably expressing HLA-A*02:01 and PPI were transduced with three different CRISPR-Cas9 vectors containing a gRNA sequence directed against the N-terminal region of the HRD1 gene and selected with puromycin. Polyclonal cell populations were analyzed for HRD1 and proinsulin levels by Western blotting. B. Monoclonal knockout cell lines were generated by limiting dilution of gRNA#1 polyclonal cells shown in (A). Genomic DNA was isolated and sequenced for the presence of deletions within the gRNA target region. Both HRD1 alleles of clones 2 and 3 were aligned to a reference sequence (NCBI gene entry 7327). C. K562 cells from (B) were retransduced with an empty cDNA vector (KO) or a cDNA vector encoding UBE2G2 (WT), or a catalytically inactive mutant (C89S). Cells were sorted on mAmetrin expression to obtain a pure population. Next cell lysates were analyzed by WB for expression of HRD1 and proinsulin. Human transferrin receptor was used as a loading control. (TIF) [file pone.0287877.s003.tif]

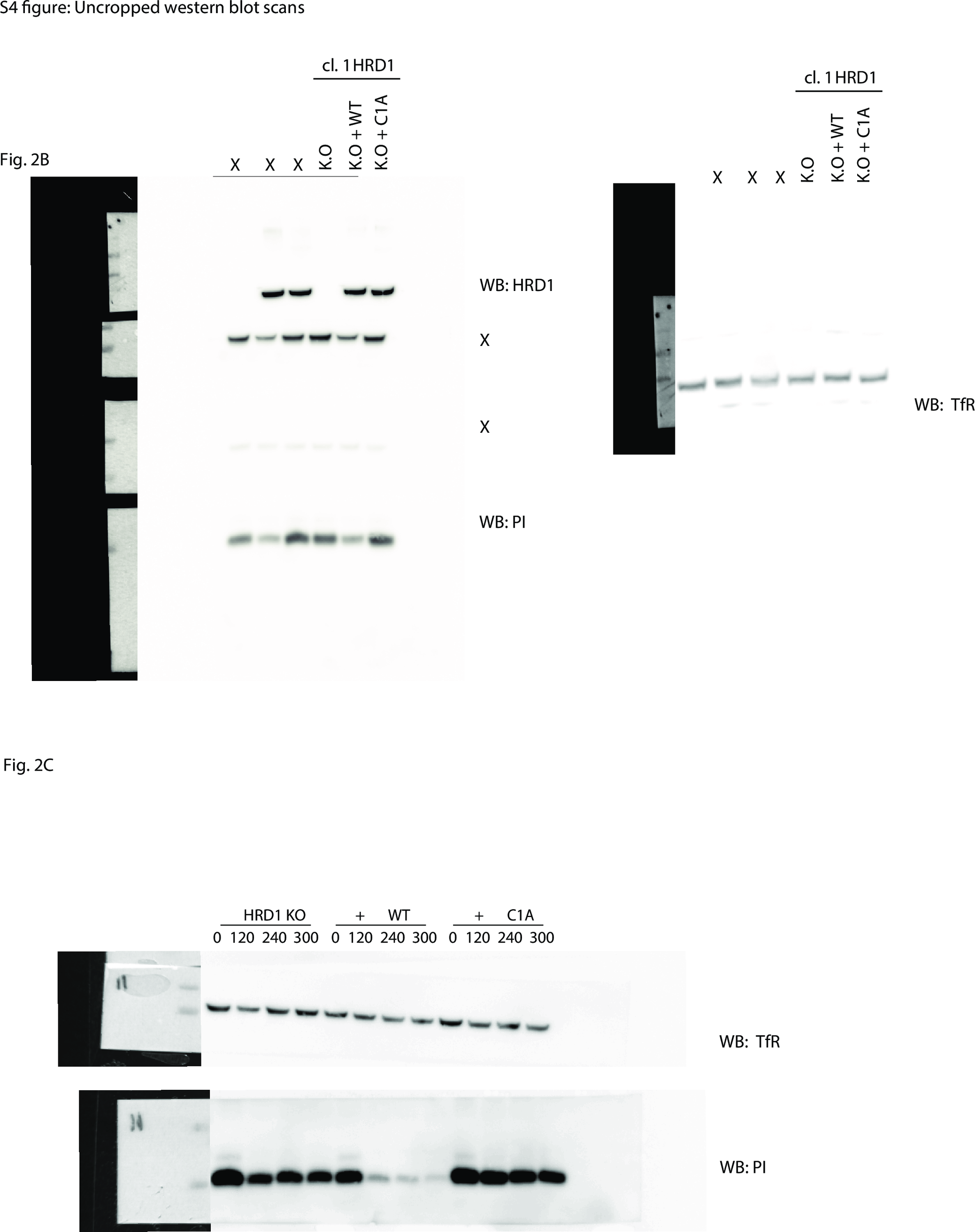

Supplement: S4 Fig — (TIF) [file pone.0287877.s004.tif]

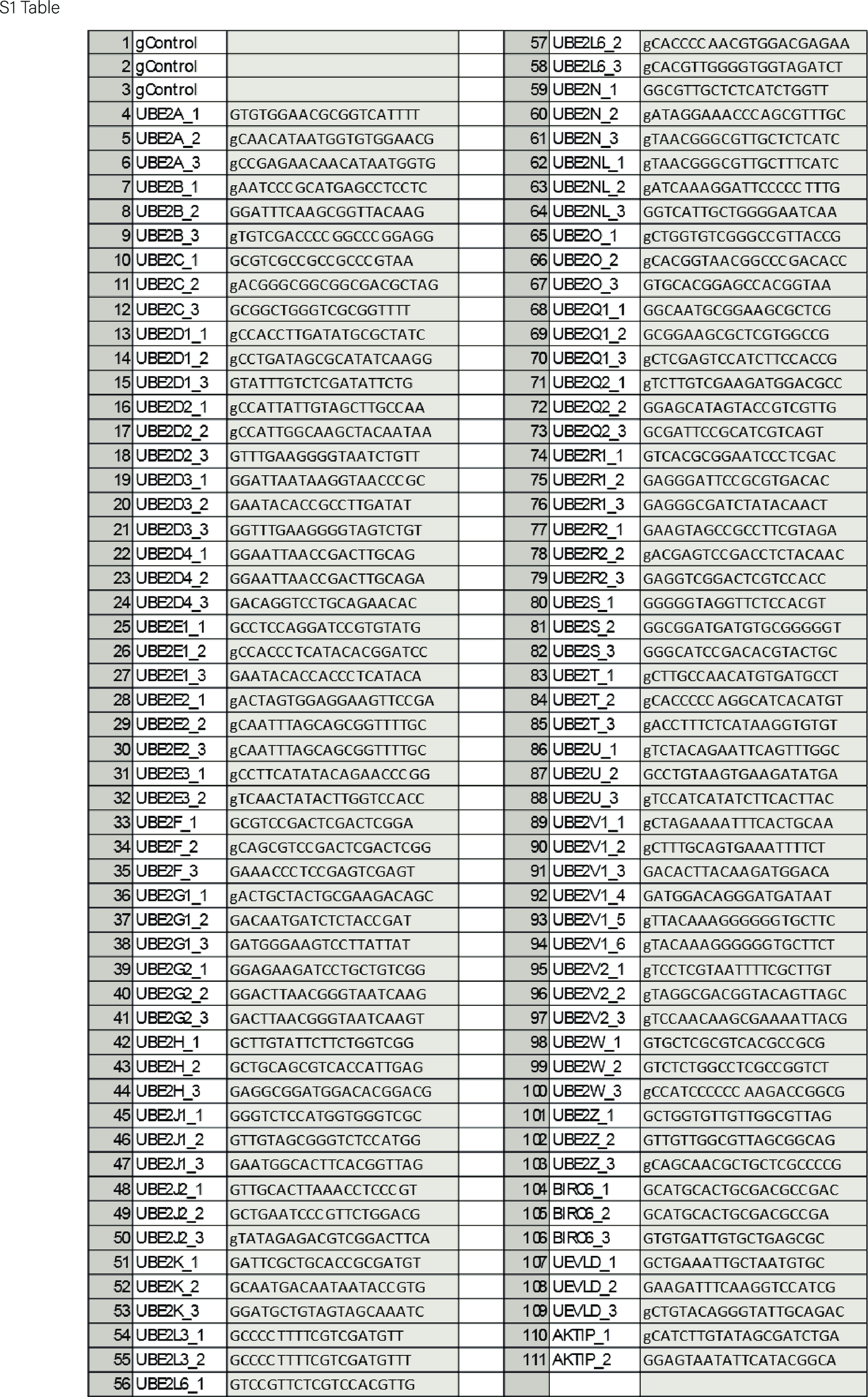

Supplement: S1 Table — (TIF) [file pone.0287877.s005.tif]

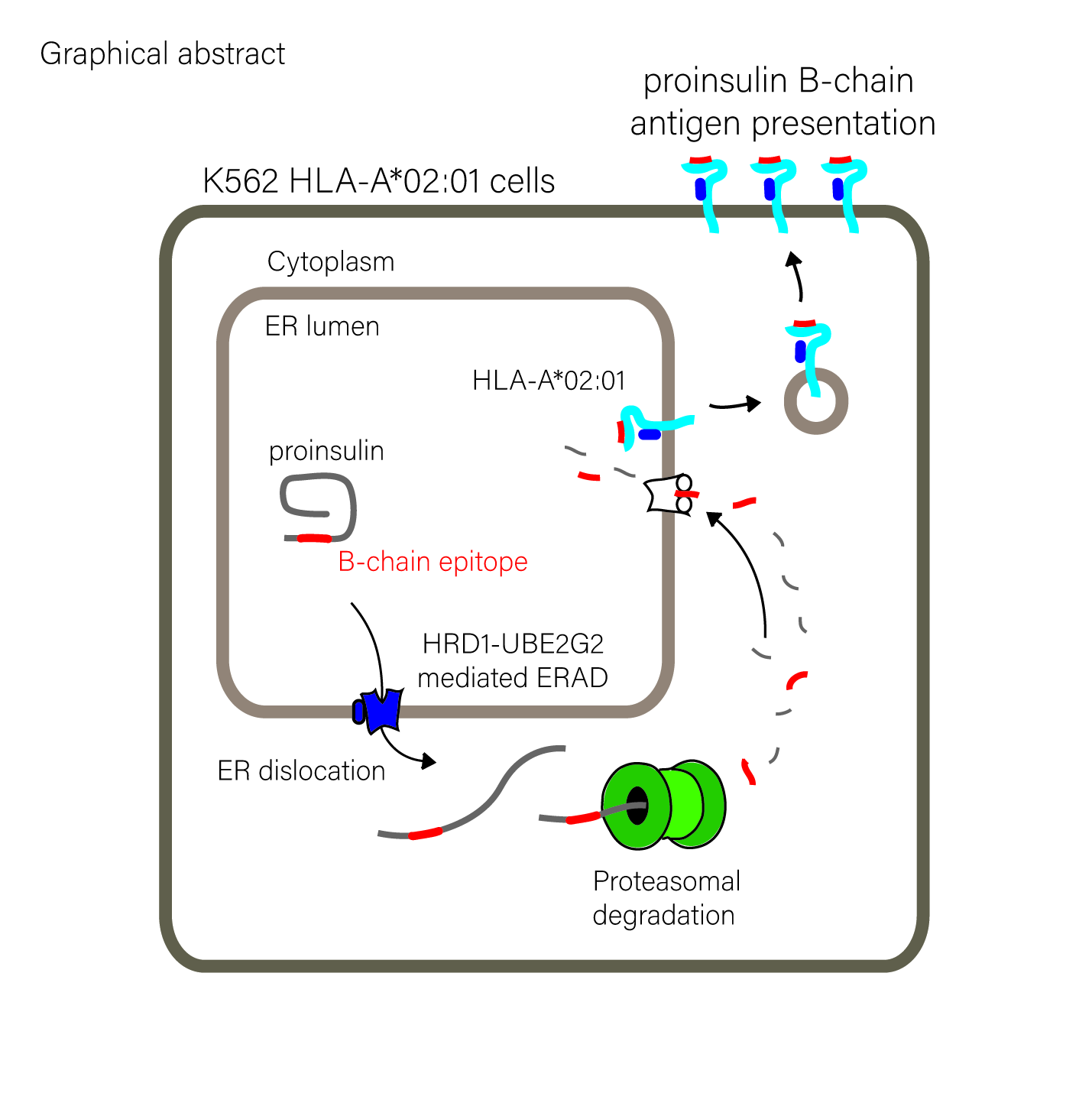

Supplement: S1 Graphical abstract — (TIF) [file pone.0287877.s008.tif]
