# Supplementary material for: Proinsulin degradation and presentation of a proinsulin B-chain autoantigen involves ER-associated protein degradation (ERAD)-enzyme UBE2G2
Source: PLoS One. 2024 May 24;19(5):e0287877. doi: 10.1371/journal.pone.0287877 (PMC11125532; doi:10.1371/journal.pone.0287877)
Supplement: Attachment — Submitted filename: Review Report_PONE-D-23-18345.pdf [file pone.0287877.s009.pdf]

In the manuscript, PONE-D-23-18345: Proinsulin degradation and presentation of a proinsulin B-chain autoantigen involves ER-associated protein degradation (ERAD)-enzyme UBE2G2, Cremer et al report UBE2G2 as a plausible Ubiquitin-conjugating enzyme (E2) that is involved in proinsulin degradation, and that the degraded products (peptides) could serve as autoantigens, especially in the context of type-1 diabetes (T1D). The experimental design, methods, and interpretation of the results are good, and the discussion is well-written. The idea of preproinsulin being a primary degradation substrate is interesting, and the results relating to antigen presentation certainly have clinical value. While K562 cells appear to be a decent model to explore intracellular events, given the premise of the study is proinsulin degradation, the reviewer strongly recommends the usage of pancreatic beta cells (cultured cell lines like INS1 or MIN6) in this study. Specific comments for the authors can be found below.

1. The authors should determine whether UBE2G2 displays the suggested function in pancreatic beta cells. Would UBE2G2 knockout or siRNA-based knockdown of endogenous UBE2G2 or similar proteins in beta cells produce a phenotype wherein the undegraded substrate (proinsulin) accumulates in cells? Please include suitable experiments to address this question. The authors may consider using wild-type or MIDY mutants of proinsulin for this study.

2. As the authors describe in the introduction, proinsulin after synthesis enters the endoplasmic reticulum (ER), gets folded, and is trafficked to Golgi and secretory granules, making insulin en route. Proinsulin that does not proceed from ER to Golgi is presumably degraded by ERAD after a retrotranslocation via the ERAD channel. Considering these are the events that are known to occur naturally, how do the authors justify the experiments, wherein the glycosylation sites are artificially introduced, possibly enhancing the half-life of preproinsulin in cells? Though previous studies using beta cells indicate that preproinsulin may serve as autoantigens, the current model and the approach used by the authors in the current work seems impracticable. Once again, the reviewer emphasizes on the utilization of beta cells.

3. The authors state that the newly-synthesized proinsulin is misfolded in K562 cells. The reviewer suggests this assertion must be substantiated with experiments. It should also be addressed whether substituting proinsulin Cys residues with other amino acids itself may drive misfolding of the protein.

4. Include a brief discussion of post-translational modifications proinsulin may undergo in the beta cells, and how these may stabilize (or destabilize) preproinsulin making it a viable degradation substrate and/or autoantigen.

5. Optional experiment: If it is possible, please show the levels or activity of UBE2G2 (or similar related protein(s) in beta cells) in purified pancreatic islets from T1D models (NOD or Akita) versus healthy controls. This would greatly enhance the physiological viewpoint of the manuscript.
