# Supplementary material for: Proinsulin degradation and presentation of a proinsulin B-chain autoantigen involves ER-associated protein degradation (ERAD)-enzyme UBE2G2
Source: PLoS One. 2024 May 24;19(5):e0287877. doi: 10.1371/journal.pone.0287877 (PMC11125532; doi:10.1371/journal.pone.0287877)
Supplement: Attachment — Submitted filename: Point-by-point response to reviewers_FINAL.pdf [file pone.0287877.s010.pdf]

Point by point response to Editor:

Below you can find detailed responses (in blue) to the points raised during the review process for the manuscript by Cremer et al., entitled "*Proinsulin degradation and presentation of a proinsulin B-chain autoantigen involves ER-associated protein degradation (ERAD)-enzyme UBE2G2*" (PONE-D-23-18345). We thank the Editor and Reviewers for their insightful commentary and constructive suggestions. We hope that all concerned deem our revision efforts compelling and look forward to your conclusions.

Editor's comments: The reviewers and myself agree that the findings are potentially of importance, but both reviewers raised very important points that need to be addressed.
In additions to their comments, I'm concerned that the low n values in some of the experiments (n=3 in Figure 2D and 4D - both of which failed to be referenced in the Results section as well) are going to make statistical significance extremely challenging unless additional repeats are provided. And while convincing, the blots in Figure 5 also lack quantification of any sort. When submitting your revision, we need you to address these additional requirements.

We sincerely apologize for omitting the statistical test results. We also noticed that the original graphs depicted mean + SEM, which does not give insight into data variation. In compliance with the editor's suggestion, we have repeated the experiments in figures 2D and 4D and have appended the additional analyses in our revised manuscript, referencing them correctly in the results section.

Considering the blots in figure 5, we have removed the conclusion on stability of the glycosylated variants from the Results section as we regard this experiment as a qualitative validation of glycosylation of our mutant proinsulin variants in cells. We hope that our results, including the thorough statistical analyses in fig. 2D and 4D, are now in compliance with research standards.

1. Please ensure that your manuscript meets PLOS ONE's style requirements, including those for file naming. The PLOS ONE style templates can be found at https://journals.plos.org/plosone/s/file?id=wjVg/PLOSOne_formatting_sample_main_body.pdf and https://journals.plos.org/plosone/s/file?id=ba62/PLOSOne_formatting_sample_title_authors_affiliations.pdf

All file names and text styles are now redone according to PLOS style.

2. We note that the grant information you provided in the 'Funding Information' and 'Financial Disclosure' sections do not match.

When you resubmit, please ensure that you provide the correct grant numbers for the awards you received for your study in the 'Funding Information' section.

We have checked the grant numbers and provided the correct ones during resubmission.

3. We note that you have stated that you will provide repository information for your data at acceptance. Should your manuscript be accepted for publication, we will hold it until you provide the relevant accession numbers or DOIs necessary to access your data. If you wish to make changes to your Data Availability statement, please describe these changes in your cover letter and we will update your Data Availability statement to reflect the information you provide.

All raw data is now provided as supplementary info.

4. PLOS ONE now requires that authors provide the original uncropped and unadjusted images underlying all blot or gel results reported in a submission's figures or Supporting Information files. This policy and the journal's other requirements for blot/gel reporting and figure preparation are described in detail at https://journals.plos.org/plosone/s/figures#loc-blot-and-gel-reporting-requirements and https://journals.plos.org/plosone/s/figures#loc-preparing-figures-from-image-files. When you submit your revised manuscript, please ensure that your figures adhere fully to these guidelines and provide the original underlying images for all blot or gel data reported in your submission. See the following link for instructions on providing the original image data: https://journals.plos.org/plosone/s/figures#loc-original-images-for-blots-and-gels.

In your cover letter, please note whether your blot/gel image data are in Supporting Information or posted at a public data repository, provide the repository URL if relevant, and provide specific details as to which raw blot/gel images, if any, are not available. E-mail us at plosone@plos.org if you have any questions.

We have included uncropped and unadjusted images of western blot data from the main figures as supplemental figures as indicated in the cover letter.

5. We note that you have included the phrase "data not shown" in your manuscript. Unfortunately, this does not meet our data sharing requirements. PLOS does not permit references to inaccessible data. We require that authors provide all relevant data within the paper, Supporting Information files, or in an acceptable, public repository. Please add a citation to support this phrase or upload the data that corresponds with these findings to a stable repository (such as Figshare or Dryad) and provide and URLs, DOIs, or accession numbers that may be used to access these data. Or, if the data are not a core part of the research being presented in your study, we ask that you remove the phrase that refers to these data.

As the experiment to which the phrase refers is not essential to the message of our manuscript, we have rephrased this sentence and excluded ''data not shown''.

6. Please include a copy of Table 1 which you refer to in your text on page 6.

We apologize for omitting this supplemental information and have included it in our resubmission.

7. Please include captions for your Supporting Information files at the end of your manuscript, and update any in-text citations to match accordingly. Please see our Supporting Information guidelines for more information.

We have transferred the captions accordingly.

Point-by-point response to reviewers:

Reviewer #1: In this manuscript, the authors seek to better understand how proinsulin is degraded by ER-associated degradation (ERAD) and whether this process contributes to generation of (potentially abnormal) peptides that are then used by HLA in antigen presentation. Understanding this process is of

clinical importance, as presentation of (abnormal) proinsulin peptides has been proposed as a trigger for autoimmune diabetes. Although the data are interesting and shown with a reasonable experimental approach, there are a few outstanding issues needed to confirm the findings summarized in the proposed model.

We thank the Reviewer for her/his appreciation of our experimental data and interest in our study; we also appreciate the constructive suggestions to improve the manuscript.

Major comments:
• No description/summary of statistical analyses; for graphs only averages +/- SD is shown (not actual values)

We sincerely apologize for omitting the summary of statistics and have provided a detailed analysis in the revised figures with appropriate descriptions in the figure legends. We decided not to show all replicate values because that would severely decrease comprehensibility of the graph but the numerical data points values are now shown as supplementary information. We hope to have addressed this issue sufficiently.

• In Figures 2 and 4, it would be helpful to show the response of true control ("EV") cells alongside the knockout and re-transduced cells. Do the knockout + HRD1/2G2 WT cells treated with cycloheximide mimic what happens in treated cells with endogenous HRD1/2G2? Or does overexpression of the replaced HRD1/2G2 itself affect proinsulin turnover?

We agree with the reviewer that ectopic overexpression of enzymes may alter downstream degradation kinetics. Considering UBE2G2, protein levels during ectopic expression were similar to those observed in the context of endogenous expression (Fig. 4C). Ectopic expression does not appreciably alter PI steady-state levels.
For HRD1, it should be taken into consideration that E3 ligase expression is often a rate-limiting step. Therefore, overexpression is expected to affect PI steady-state levels and degradation kinetics, which was previously shown for other HRD1 substrates [1]. However, the primary aim of our CHX chase experiments was to assess the effect of E2 or E3 enzymatic activity on proinsulin degradation kinetics within the same clonal setting, and therefore have excluded the heterogeneous parental population from these experiments. Therefore, we reconstituted the WT or catalytically inactive enzymes to comparable levels, which allows specific measurement of the influence of HRD1 and UBE2G2 catalytic residues on proinsulin degradation and excludes the influence of potential overexpression effects on this conclusion. We thank the reviewer for this comment and hope to have addressed it sufficiently.

• Were the (N-glycosylated mutant) proinsulin forms transfected into HRD1 KO cells? This is an important experimental control to show that HRD1-mediated ERAD itself is necessary to generate the proinsulin peptides that are later presented by HLA.

We thank the reviewer for suggesting this important control experiment. As a follow-up experiment, we performed mass spectrometry on the eluted HLA peptidome of our set of (reconstituted) HRD1 KO cell lines and manually quantified the peak height, comparing them to control parental K562-A2*01 cells. In HRD1 KO cells or cells expressing the catalytically inactive HRD1 protein, we observed a dramatic decrease in the number of peptides derived from the proinsulin B-chain. However, we also observed a decrease in cell surface presented peptides as a function of HRD1 activity (see Fig. R1). Thus, while HRD1 activity seems to be important for proinsulin degradation, it also seems to affect HLA*A2-dependent antigen presentation in general. Although interesting, these data describing a general effect of HRD1 ablation on antigen presentation is out of the scope of our manuscript and therefore we decided not to include these in our revised version.
We acknowledge that we cannot directly link HRD1 and UBE2G2 activity to presentation of proinsulin-

derived peptides although we point out that this requires dislocation over the ER membrane via ERAD, of which HRD1 and UBE2G2 are core enzymes.

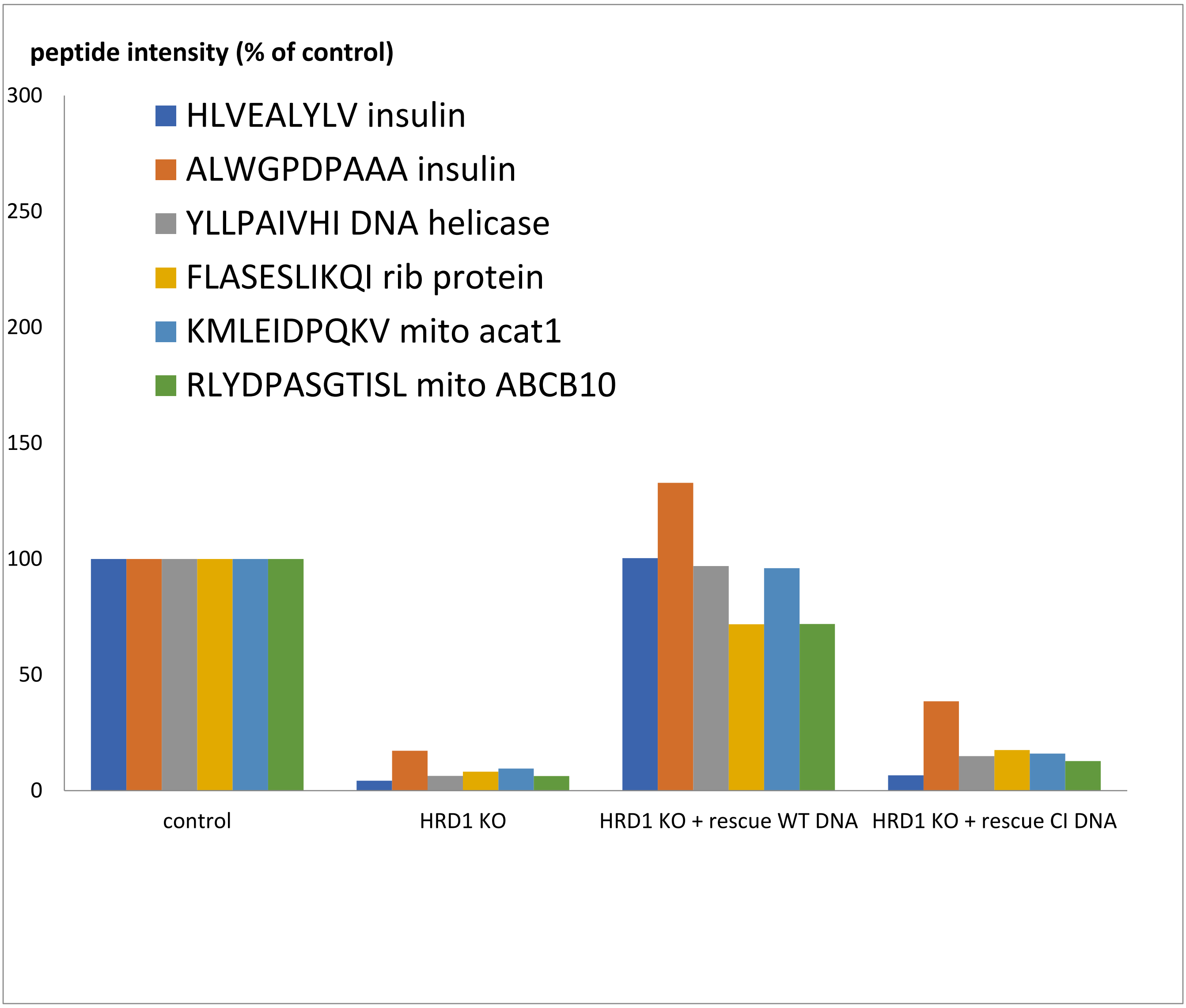

Figure R1: presentation of a proinsulin B-chain peptide and other HLA-A2*01 bound peptides require HRD1 catalytic activity. Eluted peptide intensities were manually quantified (peak height) and normalized to parental K562-A2-PPI cells (control)

• There was little introduction to the role of TAP in the antigen presentation process here. Is TAP required for ER uptake and attachment of substrates to the HLA molecules that are presenting the proinsulin peptides, or are there other mechanisms? It would be helpful to show that TAP knockout abolishes uptake and association of the mutant proinsulin peptides with HLA, as proposed in Figure 1.

We thank the reviewer for pointing out this confusing issue. Indeed, TAP-deficient individuals do not completely lack CD8-responses, pointing at TAP-independent mechanisms of peptide import into the ER [2]. Also, proinsulin-derived peptides such as signal-sequence-derived peptides [3] can be presented by TAP-independent mechanisms. These nuances have been addressed in our revised manuscript **(lines 87-88).** Although we agree it would be interesting to verify the role of TAP in the loading of proinsulin

antigens on HLA class I, our study focuses on processes that occur prior to ER import of peptides. Therefore, we believe that experiments on the role of TAP in proinsulin presentation are out of the scope of this manuscript.

Minor comments:

• On line 200, text should refer to Figure 2D, not 1D

• For Figure 2D, would recommend using similar labeling as Figure 4D – for example, I believe the authors are referring to “HRD1 KO” cells instead of “EV” as shown

We thank the reviewer for these minor comments and have addressed these in the revised manuscript.

• Frequent switching between “(pre)proinsulin” and "PPI”/”PI” makes the text difficult to read at times.

We agree that nomenclature on proinsulin can be confusing at times. For clarity, we use preproinsulin (PPI) in the context of DNA/RNA and peptides derived from the unprocessed form of insulin, while PI is used for proinsulin molecules from which the signal peptide has been removed after entering the ER. We have clarified the use of these abbreviations in our revised introduction.

Reviewer #2:

In the manuscript, PONE-D-23-18345: Proinsulin degradation and presentation of a proinsulin B-chain autoantigen involves ER-associated protein degradation (ERAD)-enzyme UBE2G2, Cremer et al report UBE2G2 as a plausible Ubiquitin-conjugating enzyme (E2) that is involved in proinsulin degradation, and that the degraded products (peptides) could serve as autoantigens, especially in the context of type-1 diabetes (T1D). The experimental design, methods, and interpretation of the results are good, and the discussion is well-written. The idea of preproinsulin being a primary degradation substrate is interesting, and the results relating to antigen presentation certainly have clinical value. While K562 cells appear to be a decent model to explore intracellular events, given the premise of the study is proinsulin degradation, the reviewer strongly recommends the usage of pancreatic beta cells (cultured cell lines like INS1 or MIN6) in this study. Specific comments for the authors can be found below.

We thank the Reviewer for her/his favorable assessment of our study and appreciate the constructive suggestions offered to improve it. We agree that expanding the results obtained in our K562 model system to the pancreatic beta cell lines recommended by the reviewer would greatly extend the clinical value of our study. However, this would also increase the complexity of the studies. We have explored the use of pancreatic beta cell lines and have run into many practical issues. We mentioned these suggestions in our Discussion section and hope that we have sufficiently addressed the limitations of our model system in our revised manuscript.

1. The authors should determine whether UBE2G2 displays the suggested function in pancreatic beta cells. Would UBE2G2 knockout or siRNA-based knockdown of endogenous UBE2G2 or similar proteins in beta cells produce a phenotype wherein the undegraded substrate (proinsulin) accumulates in cells? Please include suitable experiments to address this question. The authors may consider using wild-type or MIDY mutants of proinsulin for this study.

We thank the reviewer for her/his suggestion. As ERAD mechanisms are conserved across species and between cell types, especially regarding soluble substrates, we believe that our findings on the role of core ERAD machinery in degradation of proinsulin can be extended to other cell types, such as pancreatic beta cells. We have addressed this suggestion in our revised Discussion section (**lines 316-317**).

2. As the authors describe in the introduction, proinsulin after synthesis enters the endoplasmic reticulum (ER), gets folded, and is trafficked to Golgi and secretory granules, making insulin en route. Proinsulin that does not proceed from ER to Golgi is presumably degraded by ERAD after a retrotranslocation via the ERAD channel. Considering these are the events that are known to occur naturally, how do the authors justify the experiments, wherein the glycosylation sites are artificially introduced, possibly enhancing the half-life of preproinsulin in cells? Though previous studies using beta cells indicate that preproinsulin may serve as autoantigens, the current model and the approach used by the authors in the current work seems impracticable. Once again, the reviewer emphasizes on the utilization of beta cells.

We agree with the reviewer that artificially introducing mutations in substrate proteins may steer away substrates from their natural degradation pathways. Mutation of cysteine residues involved in disulfide bond formation, like C31, would indeed preclude the successful folding of proinsulin molecules and affect their stability. However, cells expressing our C96N mutant also present the natural B5-14 epitope, indicating that it does not affect the dislocation of the protein as it still accesses cytosolic proteasomes that generate the same B5-14 epitope. We therefore believe that it is safe to conclude that the biochemical pathways that generate this specific antigen are still used as natural, i.e. dislocation over the ER membrane into the cytosol and peptide generation by the proteasome. In our revised manuscript, we specify our claims only regarding this this antigen specifically.

3. The authors state that the newly-synthesized proinsulin is misfolded in K562 cells. The reviewer suggests this assertion must be substantiated with experiments. It should also be addressed whether substituting proinsulin Cys residues with other amino acids itself may drive misfolding of the protein.

We thank the reviewer for her/his feedback on our Discussion section. We agree that the substitution of proinsulin Cys molecules likely affects protein folding and stability, but the results from our C96N mutant suggest that this does not qualitatively alter the presentation of the PPI $B_{5\text{-}14}$ epitope. We have discussed this in more detail in our revised manuscript **(lines 296-298)**. We have also removed the statement about misfolding of WT proinsulin in our model system as we do not provide data that proves this.

4. Include a brief discussion of post-translational modifications proinsulin may undergo in the beta cells, and how these may stabilize (or destabilize) preproinsulin making it a viable degradation substrate and/or autoantigen.

We appreciate the suggestion to discussthe details of proinsulin stability through post-translational modification and now mention the specifics of proinsulin ubiquitination in our revised manuscript **(lines 307-309).** Furthermore, we have expanded our discussion on proinsulin stability through disulfide bond formation and how our glycosylated mutants may affect this (**lines 293-295)**.

5. Optional experiment: If it is possible, please show the levels or activity of UBE2G2 (or similar related protein(s) in beta cells) in purified pancreatic islets from T1D models (NOD or Akita) versus healthy controls. This would greatly enhance the physiological viewpoint of the manuscript.
We thank the reviewer for their excellent suggestions for extra experiments. Expanding our findings in beta cells would indeed greatly enhance the study from a physiological viewpoint. We have added the suggestion for this experiment in our revised Discussion section **(lines 317-319).**

References

1. Liu L, Long H, Wu Y, Li H, Dong L, Zhong JL, et al. HRD1-mediated PTEN degradation promotes cell proliferation and hepatocellular carcinoma progression. Cell Signal. 2018;50:90-9. doi: 10.1016/j.cellsig.2018.06.011. PubMed PMID: 29958993.
2. De la Salle H, Saulquin X, Mansour I, Klayme S, Fricker D, Zimmer J, et al. Asymptomatic deficiency in the peptide transporter associated to antigen processing (TAP). Clinical & Experimental Immunology. 2002;128(3):525-31.
3. Kronenberg-Versteeg D, Eichmann M, Russell MA, de Ru A, Hehn B, Yusuf N, et al. Molecular Pathways for Immune Recognition of Preproinsulin Signal Peptide in Type 1 Diabetes. Diabetes. 2018;67(4):687-96. doi: 10.2337/db17-0021. PubMed PMID: 29343547.
