# Supplementary material for: Proinsulin degradation and presentation of a proinsulin B-chain autoantigen involves ER-associated protein degradation (ERAD)-enzyme UBE2G2
Source: PLoS One. 2024 May 24;19(5):e0287877. doi: 10.1371/journal.pone.0287877 (PMC11125532; doi:10.1371/journal.pone.0287877)
Supplement: Attachment — Submitted filename: Response to reviewers after resubmission.docx [file pone.0287877.s011.docx]

**Response to reviewers after resubmission**

*Editor:* I went over the manuscript and review again.

Both reviewers agreed that experiments assessing a reduction in UBE2G2 would be central to ascertain the conclusions of the manuscript.

Reviewer #2 summarizes my thought on the matter really well and ends with:

“Please note that the reviewer is not insisting on using purified islets from diabetes models but suggesting experiments using beta cell lines (MIN6 or INS1).”

The request doesn’t seem unreasonable or incredibly time consuming if performed on cell lines and would answer all concerns raised but both reviewers 1 and 2. It is also not incredibly technically challenging and I suspect even a graduate student should be able to at least test UBE2G2 inhibition using a CW3 compound.

That being said, I’m not unreasonable and I understand the circumstances. If there is truly no way these experiments can be performed considering the context, they should resubmit the manuscript rewriting the conclusion (and they should explain to the reviewers why they can’t do the experiments).

We are grateful for the flexibility of the editor and understand the concerns raised by the reviewers. To meet the request of reviewer#1, we have now appended the previously performed quantitative mass spectrometry experiments to our manuscript, including highly suggestive data towards a specific role of UBE2G2 in proinsulin antigen presentation. However, for reasons communicated before, we are unable to perform any additional experiments requested by reviewer#2. Nevertheless, we hope to publish our manuscript with PLOS ONE with revised conclusions. T

*Reviewer #1:* Most of my concerns have been sufficiently addressed. However, I believe the manuscript’s conclusion is still stated too strongly based on the data presented. I appreciate the authors’ attempt to show that generation of proinsulin-derived peptides in HRD1 KO cells is greatly reduced compared to controls (Figure R1), and understand their interpretation that the concomitant reduction in antigen presentation capacity in the HRD1 KO cells confounds the conclusion that HRD1 inactivation directly affects proinsulin-derived peptide antigen presentation. As stated in the response, “We acknowledge that we cannot directly link HRD1 and UBE2G2 activity to presentation of proinsulin-derived peptides although we point out that this requires dislocation over the ER membrane via ERAD, of which HRD1 and UBE2G2 are core enzymes.” However, this leaves little data presented to link the role of HRD1 ERAD (with UBE2G2) in stabilizing wild-type proinsulin to the observed findings that mutated proinsulin forms appear to be shuttled to the cytoplasm, where they are modified before antigen presentation. Since using HRD1 KO cells in the experiments shown in Figure 6 may lead to confounded results, the experiment could be repeated with UBE2G2 KO cells – and according to the proposed model, there should be a reduction in modified epitopes detected compared to using cells with functional UBE2G2-HRD1 ERAD. If this experiment cannot be accomplished, the conclusions should be modified to deemphasize the direct link as proposed in the graphical abstract and throughout the Discussion section (for example, lines 350-352, “Our data strongly suggests that degradation of proinsulin through HRD1 and UBE2G2-mediated ERAD results in presentation of a proinsulin B-chain autoantigens.”). Alternatively, adding Figure R1 to the manuscript with a brief discussion of the conclusions from that experiment would be helpful to explain why the direct experiment is not possible.

We thank the reviewer for his suggestion to include the quantitative peptidome analysis in our manuscript. Previously, we have also performed the experiment in the (rescued) UBE2G2 KO cell panel, and are pleased to report its reanalysis upon request of the reviewer. The data in Fig. 6E suggests a specific role for this enzyme in the presentation of a PPI-derived B-chain autoantigen, while the previously communicated data in Fig. 4E (HRD1 KO) suggests a general role for HRD1 in antigen presentation in our model system. In line with our model, these data link UBE2G2 to proinsulin antigen presentation. We believe that the full dataset (including the HRD1 KO series) is of great value to the scientific community and will share it as a supplemental table in our manuscript. All spectra are uploaded to the PRIDE platform.

*Reviewer #2:* No experiments suggested by the reviewer have been performed. While the authors intend to do additional relevant work in the future, the reviewer again emphasizes that the UBE2G2 function in the pancreatic beta cells should be determined as part of this manuscript. Does UBE2G2 knockdown, knockout, or functional inhibition (using CW3 compound, for example) in the beta cells lead to the accumulation of undegraded proinsulin? Please note that the reviewer is not insisting on using purified islets from diabetes models but suggesting experiments using beta cell lines (MIN6 or INS1). Without these studies, the data in the manuscript does not satisfactorily support the conclusions.

We thank the reviewer for his/her suggestions to improve our manuscript by performing additional experiments in mouse beta cell lines. However, since the lab has been discontinued and all persons involved have been involved in different projects for some time now, we are not able to set up these experiments. Nevertheless, we have discussed the value of these experiments in our discussion section and have revised the relevant conclusions throughout the text, and emphasized the use of our K562 model in our graphical abstract.
